# Association between high-flow nasal cannula use and mortality in patients with sepsis-induced acute lung injury: a retrospective propensity score-matched cohort study

**DOI:** 10.1186/s12890-024-03022-9

**Published:** 2024-04-22

**Authors:** Lijun Song, Min Li, Tianlong Zhang, Lei Huang, Jianjun Ying, Lan Ying

**Affiliations:** 1 Department of Critical Care Medicine, The Fourth Affiliated Hospital, Zhejiang University School of Medicine, Yiwu, Zhejiang China; 2 Department of General Medicine, Yiwu Traditional Chinese Medicine Hospital, Yiwu, Zhejiang China; 3 Department of Emergency Medicine, Second Affiliated Hospital, Zhejiang University School of Medicine, Hangzhou, Zhejiang China

**Keywords:** High-flow nasal cannula, Sepsis, Acute lung injury, Mortality, MIMIC-IV, Propensity score matching

## Abstract

**Background:**

High-flow nasal cannula (HFNC) has emerged as a promising noninvasive method for delivering oxygen to critically ill patients, particularly those with sepsis and acute lung injury. However, uncertainties persist regarding its therapeutic benefits in this specific patient population.

**Methods:**

This retrospective study utilized a propensity score-matched cohort from the Medical Information Mart in Intensive Care-IV (MIMIC-IV) database to explore the correlation between HFNC utilization and mortality in patients with sepsis-induced acute lung injury. The primary outcome was 28-day all-cause mortality.

**Results:**

In the propensity score-matched cohort, the 28-day all-cause mortality rate was 18.63% (95 out of 510) in the HFNC use group, compared to 31.18% (159 out of 510) in the non-HFNC group. The use of HFNC was associated with a lower 28-day all-cause mortality rate (hazard ratio [HR] = 0.53; 95% confidence interval [CI] = 0.41–0.69; *P* < 0.001). HFNC use was also associated with lower ICU mortality (odds ratio [OR] = 0.52; 95% CI = 0.38–0.71; *P* < 0.001) and lower in-hospital mortality (OR = 0.51; 95% CI = 0.38–0.68; *P* < 0.001). Additionally, HFNC use was found to be associated with a statistically significant increase in both the ICU and overall hospitalization length.

**Conclusions:**

These findings indicate that HFNC may be beneficial for reducing mortality rates among sepsis-induced acute lung injury patients; however, it is also associated with longer hospital stays.

**Supplementary Information:**

The online version contains supplementary material available at 10.1186/s12890-024-03022-9.

## Introduction

In recent years, HFNC has emerged as a notable noninvasive method for delivering highly concentrated oxygen, particularly in patients who are experiencing challenges in standard oxygen therapy management [1–3]. With the capacity to supply an airflow ranging from 50–60 L/min [4], HFNC ensures consistent oxygen concentrations while applying low positive end-expiratory pressure in the upper airway [3], thereby significantly alleviating the respiratory effort required by the patient. Numerous guidelines and studies affirm the favorable therapeutic impact of HFNC on hypoxic respiratory failure, reducing the need for tracheal intubation and demonstrating a beneficial role in preventing reintubation [5–7].

Previous studies have primarily emphasized the use of HFNC in pediatric cases [8], chronic obstructive pulmonary disease [9], obstructive sleep apnea syndrome [10, 11], and post-cardiothoracic surgery patients [12], with a wealth of evidence supporting its therapeutic benefits in these specific circumstances. However, uncertainties persist regarding the therapeutic benefits of HFNC in patients with sepsis-induced acute lung injury, a condition associated with high morbidity rates in the intensive care unit (ICU). Consequently, this study delves into the correlation between HFNC utilization and mortality in patients presenting with sepsis and acute lung injury. Leveraging the Medical Information Marketplace in Intensive Care-IV (MIMIC-IV) database, we aimed to shed light on the potential impact of HFNC therapy in this critical patient population.

## Methods

### Data source

Utilizing a propensity score-matched cohort obtained from the MIMIC-IV database, a retrospective study was conducted. MIMIC-IV version 2.2 is a comprehensive electronic health record dataset developed and managed by the Massachusetts Institute of Technology's Laboratory of Computational Physiology [13]. It encompasses data from over 50,000 patients admitted to the Beth Israel Deaconess Medical Center. The Institutional Review Board, responsible for overseeing data publication, granted waivers for informed consent and approved the sharing of research resources. One of our team's authors (SLJ) obtained access to the database (certification number 59010484).

### Study population

The study population comprised adult critically ill patients diagnosed with sepsis and acute lung injury. The definition of sepsis followed the criteria outlined in the Third International Consensus Definition of Sepsis and Septic Shock (Sepsis-3) [14], which necessitates a suspected or documented infection and a minimum increase of 2 points in the Sequential Organ Failure Assessment (SOFA) score [15]. Determination of the presence of infection based on culture records and antibiotic use records. Acute lung injury was defined as an oxygenation index (the ratio of partial pressure of arterial oxygen to partial pressure of inspired oxygen, PaO_2_/FiO_2_) of less than 300 mm Hg [16]. We excluded patients who were younger than 18 years of age, admitted to the ICU for less than 24 h, had missing data on mechanical ventilation, or did not have acute lung injury after the diagnosis of sepsis. Additionally, only the initial ICU record from the first admission was incorporated into the analysis.

### Exposure and outcomes

The use of HFNC in the ICU was defined as exposure without any limitations. HFNC data were obtained from the ventilation table, and patients with incomplete HFNC exposure data were excluded from the analyses. The primary outcome was 28-day all-cause mortality. Secondary outcomes included ICU mortality, in-hospital mortality, length of stay in the ICU, and overall length of hospital stay.

### Data collection

Data extraction was carried out using PostgreSQL and Navicat Premium software (version 16.3) by executing the Structured Query Language (SQL). The SQL script code was sourced from the GitHub repository at https://github.com/MIT-LCP/mimic-iv. Demographic characteristics of patients, such as age, gender, race, ICU type, body mass index (BMI), and Charlson co-morbidity index, were collected. Treatment records subsequent to sepsis diagnosis were also extracted, encompassing 6-h and 24-h antibiotic therapy, continuous renal replacement therapy (CRRT), vasoactive medications (dopamine, epinephrine, norepinephrine, phenylephrine, pressor, dobutamine, milrinone), and mechanical ventilation data. Comorbidity information was obtained utilizing the international classification of diseases coding system, covering conditions such as cerebrovascular disease, dementia, rheumatic disease, congestive heart failure, chronic lung disease, diabetes mellitus without complications, diabetes mellitus with complications, renal disease, mild and severe liver disease, and malignancy. Additionally, initial records at the onset of sepsis were extracted, including disease severity assessed by the SOFA score, vital signs (heart rate, mean arterial pressure, respiratory rate, temperature, arterial blood oxygenation index), and laboratory investigations (white blood cell, platelet, hemoglobin, PH, lactate).

### Statistical analysis

In this study, the cohort was divided into two groups: the HFNC-treated group and the non-HFNC-treated group. To address missing data, the researchers utilized the R package "mice" for multiple imputation [17, 18], filling in missing values for each variable. The missing rates for each variable are detailed in the Supplementary Material: Table S1. To ensure the reliability of the analysis, the researchers assessed multicollinearity between variables using variance inflation factors (VIFs), calculated with the R package "car." Variables with VIF greater than 5 were excluded, as indicated in the Supplementary Material: Tables S2 and S3.

Continuous variables are presented as mean (standard deviation [SD]) or median (interquartile range [IQR]) and analyzed using appropriate statistical tests depending on the normality of the distribution. Specifically, the Student's t-test for independent samples or the Mann–Whitney U test was employed. Categorical variables are expressed as numbers and percentages and analyzed using the chi-square test or Fisher's exact test.

For the primary outcome of 28-day all-cause mortality, Cox proportional hazards models were developed, providing HR and 95% CI. Kaplan–Meier survival analysis was used to assess the incidence of endpoints in different treatment groups, and differences were assessed by log-rank tests.

Dichotomous secondary outcomes were analyzed using logistic regression models to calculate OR and 95% CI. For continuous secondary outcomes, the Hodges-Lehmann estimator was applied to calculate median differences (MDs) and 95% CI. All analyses were considered statistical significance at a two-tailed *P*-value less than 0.05. The statistical software R (version 4.2.0) was employed for conducting these analyses.

### Propensity score matching

In the preliminary analysis of the matched cohort, the researchers aimed to explore the association between HFNC use and both primary and secondary outcomes. To address potential confounding factors, propensity score matching was employed [19]. The selected variables for matching were based on consensus statements found in the literature [20], encompassing age, sex, race, ICU type, BMI, SOFA score, and arterial blood oxygenation index.

Matching was performed in a 1:1 ratio using the nearest-neighbor method, with a caliper width of 0.05 and no replacement. The balance of variables between the HFNC-treated and non-HFNC-treated groups before and after matching was assessed using the standard mean difference (SMD), with values less than 0.10 indicating a balanced distribution of variables between the groups. Specific code for calculating propensity score matching was listed in Additional file 2.

In the paired cohort dataset, variables with a significance level of *P* < 0.05 in univariate analyses were included in multivariate analyses for adjustment. These variables included cerebrovascular disease, congestive heart failure, chronic lung disease, renal disease, diabetes mellitus with complications, diabetes mellitus without complications, 24-h vasoactive medication use, 24-h mechanical ventilation recordings, mean hemoglobin, mean pH, and mean lactate levels, as detailed in the Supplementary Material: Table S4.

### Subgroup analyses

The cohorts were stratified into subgroups based on specific demographic and clinical characteristics. The defined subgroups included age stratification into < 65 years versus ≥ 65 years, gender categorization into female versus male, division of the SOFA score into < 4 versus ≥ 4, and classification of the arterial oxygenation index into subgroups of < 100 versus 100–300.

## Results

### Patient selection

**Fig. 1:**
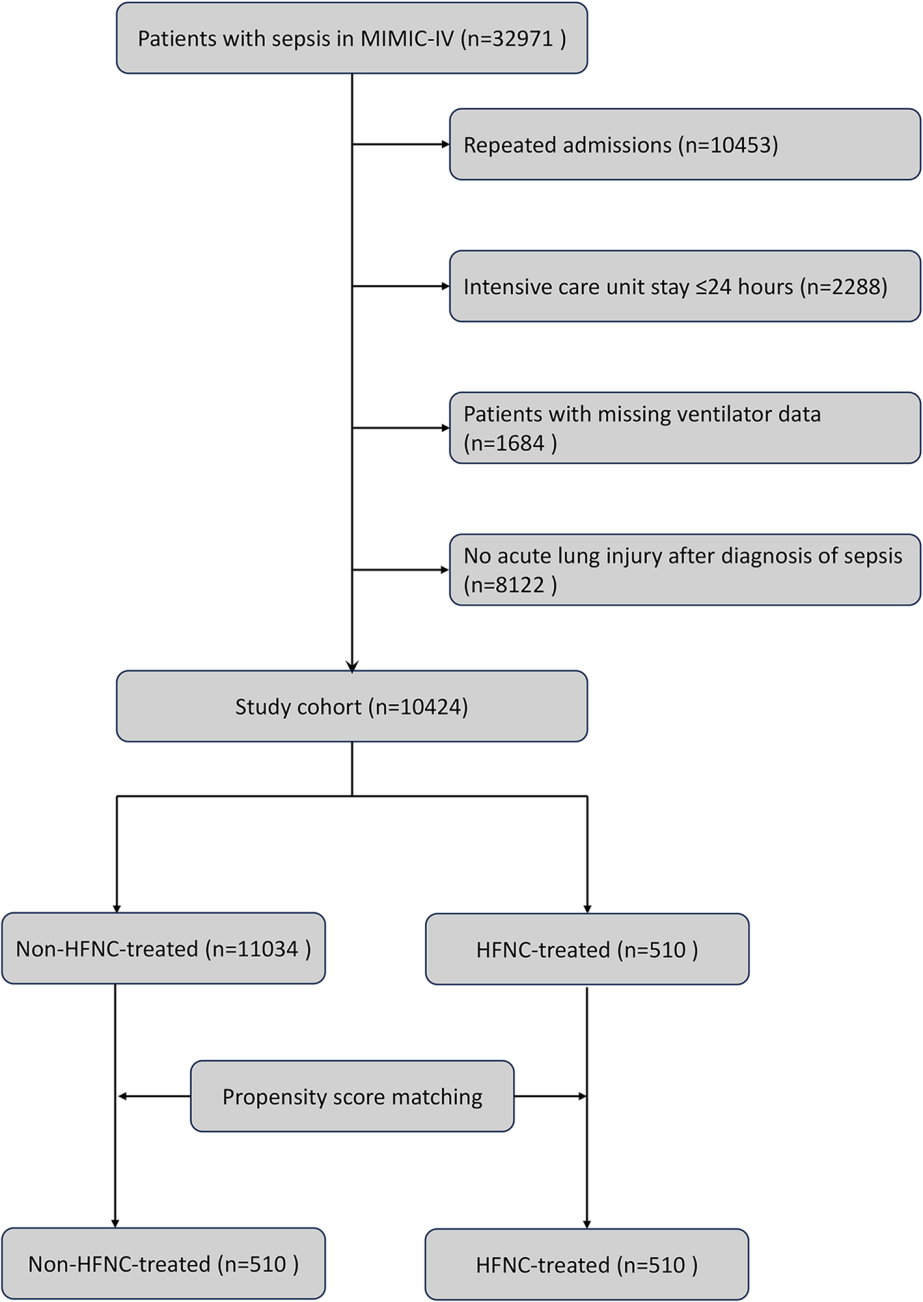
Flow chart illustrating the process of patient selection in MIMIC-IV. MIMIC-IV, the Medical Information Mart in Intensive Care-IV

In Fig. 1, the stepwise patient selection process is illustrated. Initially, a total of 32,971 records with a diagnosis of sepsis were identified. After excluding records that did not meet the eligibility criteria, a final cohort of 10,424 patients was established, among whom 510 patients received HFNC treatment during their ICU stay. The subsequent creation of a matched cohort involved 1,020 patients, with 510 individuals in each group, ensuring a balanced comparison.

### Cohort characteristics

Table 1 provides a comprehensive overview of the baseline characteristics before and after the matching process. Within the entire cohort, it was observed that patients undergoing HFNC treatment exhibited poorer arterial oxygenation index and faster respiratory rates, as indicated in the Supplementary Material: Table S4. The matching procedure significantly enhanced the variable balance, with absolute SMD below 0.10. Despite these improvements, some imbalances persisted in variables not selected for propensity score matching, as detailed in Table 1. The distribution balance, both before and after propensity score matching, is visually presented in the Supplementary Material: Figure S1.

**Table 1:** Comparison of baseline data before and after propensity score matching

Categories	Before propensity score matching			After propensity score matching		
	**No-HFNC**	**HFNC**	**SMD**	**No-HFNC**	**HFNC**	**SMD**
Number of patients	9914	510		510	510	
Age	65.71 (15.22)	65.97 (15.43)	0.017	64.60 (16.59)	65.97 (15.43)	0.086
Gender						
Female	3773 (38.1)	219 (42.9)	0.1	219 (42.9)	219 (42.9)	< 0.001
Male	6141 (61.9)	291 (57.1)		291 (57.1)	291 (57.1)	
Race						
Black	616 (6.2)	18 (3.5)	0.143	8 (1.6)	18 (3.5)	0.132
White	6619 (66.8)	344 (67.5)		343 (67.3)	344 (67.5)	
Other	950 (9.6)	44 (8.6)		51 (10.0)	44 (8.6)	
Unknow	1729 (17.4)	104 (20.4)		108 (21.2)	104 (20.4)	
First care unit						
Surgical ICU	1451 (14.6)	96 (18.8)	0.264	85 (16.7)	96 (18.8)	0.085
Medical ICU	1808 (18.2)	102 (20.0)		116 (22.7)	102 (20.0)	
CCU	4153 (41.9)	151 (29.6)		156 (30.6)	151 (29.6)	
Other	2502 (25.2)	161 (31.6)		153 (30.0)	161 (31.6)	
BMI	29.63 (7.74)	29.51 (8.63)	0.015	29.77 (8.06)	29.51 (8.63)	0.032
SOFA	3.00 [2.00, 5.00]	3.00 [2.00, 5.00]	0.045	3.00 [2.00, 5.00]	3.00 [2.00, 5.00]	0.092
Comorbidities						
Cerebrovascular disease	1496 (15.1)	58 (11.4)	0.11	87 (17.1)	58 (11.4)	0.163
Dementia	233 (2.4)	16 (3.1)	0.048	15 (2.9)	16 (3.1)	0.011
Rheumatic disease	332 (3.3)	13 (2.5)	0.047	20 (3.9)	13 (2.5)	0.078
Congestive heart failure	2915 (29.4)	180 (35.3)	0.126	142 (27.8)	180 (35.3)	0.161
Chronic pulmonary disease	2777 (28.0)	195 (38.2)	0.219	147 (28.8)	195 (38.2)	0.2
Diabetes with cc	756 (7.6)	70 (13.7)	0.199	29 (5.7)	70 (13.7)	0.274
Diabetes without cc	2452 (24.7)	103 (20.2)	0.109	135 (26.5)	103 (20.2)	0.149
Renal disease	1835 (18.5)	102 (20.0)	0.038	89 (17.5)	102 (20.0)	0.065
Mild liver disease	1469 (14.8)	76 (14.9)	0.002	81 (15.9)	76 (14.9)	0.027
Severe liver disease	678 (6.8)	42 (8.2)	0.053	39 (7.6)	42 (8.2)	0.022
Malignant cancer	1045 (10.5)	73 (14.3)	0.115	56 (11.0)	73 (14.3)	0.1
Treatment						
Antibiotic_6h	6049 (61.0)	335 (65.7)	0.097	315 (61.8)	335 (65.7)	0.082
Antibiotic_24h	7965 (80.3)	424 (83.1)	0.072	435 (85.3)	424 (83.1)	0.059
CRRT_24h	347 (3.5)	22 (4.3)	0.042	16 (3.1)	22 (4.3)	0.062
Vasoactive_24h	5954 (60.1)	247 (48.4)	0.235	315 (61.8)	247 (48.4)	0.271
Ventilation_24h	4987 (50.3)	195 (38.2)	0.245	227 (44.5)	195 (38.2)	0.128
Vital signs						
Heart rate (beats/min)	87.10 (15.53)	89.04 (16.56)	0.121	89.25 (16.46)	89.04 (16.56)	0.013
Respiratory rate (bpm)	19.97 (4.18)	21.24 (4.17)	0.305	21.09 (4.52)	21.24 (4.17)	0.033
Temperature (℃)	36.99 (0.68)	37.01 (0.54)	0.034	36.98 (0.72)	37.01 (0.54)	0.045
Mean arterial pressure (mm Hg)	75.77 (9.29)	76.44 (9.59)	0.072	75.62 (9.75)	76.44 (9.59)	0.085
Laboratory test						
WBC (10^9^/L)	12.95 (8.52)	13.76 (9.38)	0.09	13.52 (10.45)	13.76 (9.38)	0.024
Hemoglobin (g/dl)	10.13 (1.60)	9.85 (1.89)	0.159	10.21 (1.80)	9.85 (1.89)	0.195
Platelet (10^9^/L)	175.00 (96.53)	179.51 (98.52)	0.046	178.31 (97.55)	179.51 (98.52)	0.012
PH	7.37 (0.07)	7.39 (0.07)	0.195	7.36 (0.08)	7.39 (0.07)	0.295
Lactate (mmol/L)	2.17 (1.82)	1.97 (1.61)	0.119	2.40 (2.18)	1.97 (1.61)	0.224
PaO_2_/FiO_2_ (mm Hg)	156.74 (68.11)	104.87 (49.37)	0.872	103.65 (49.83)	104.87 (49.37)	0.025

*Antibiotic_6h* Antibiotics within 6 h of sepsis diagnosis, *Antibiotic_24h* Antibiotics within 24 h of sepsis diagnosis, *SMD* Standardized Mean Difference, *CCU* Cardiovascular Care Unite, *BMI* Body Mass Index, *SOFA* Sequential Organ Failure Assessment, *Diabetes with cc* diabetes mellitus with complications, *Diabetes without cc* diabetes without complications, *CRRT* Continuous Renal Replacement Therapy, *WBC* White Blood Cell, *PH* potential of hydrogen, *HFNC* High-flow nasal cannula

In the entire cohort of 10,424 patients, 510 individuals received HFNC treatment, accounting for 4.89% (510/10,424) of the total population. Following the matching process that resulted in a cohort of 1,020 patients (510 in each group), a comparison of the HFNC and non-HFNC groups revealed no statistically significant difference in the arterial oxygenation index (104.87 vs. 103.65, respectively; *P* = 0.693). Furthermore, the median duration of HFNC use within the matched cohort was reported as 15.0 h, with an IQR of 8.0 to 26 h. Notably, the duration of HFNC use varied widely, ranging from the shortest recorded duration of 0.5 h to the longest duration of 261 h.

### Primary outcome

**Fig. 2:**
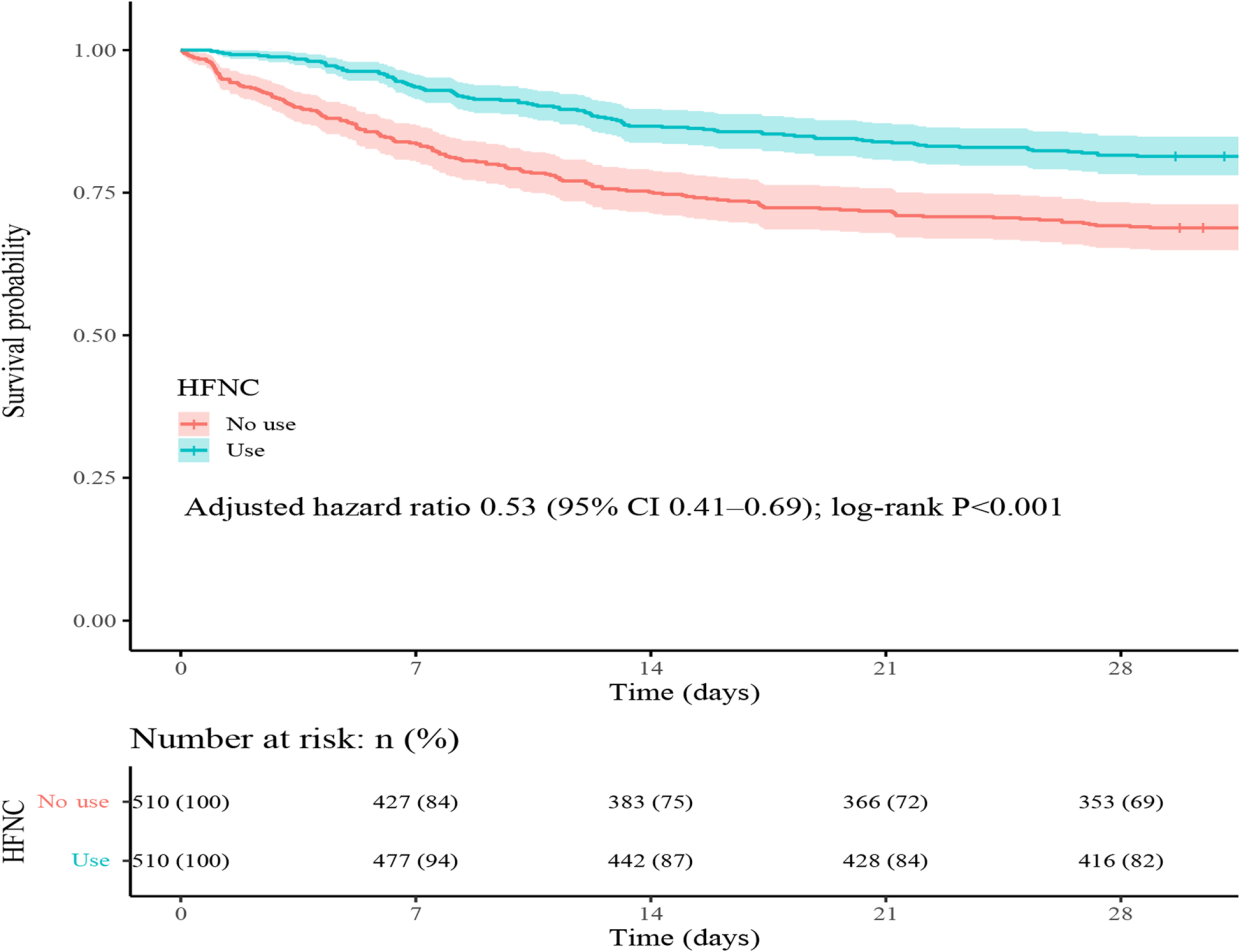
Kaplan–Meier curves for 28-day all-cause mortality based on HFNC usage in paired cohort. In the multivariable Cox proportional hazards model, adjustments were made for cerebrovascular disease, congestive heart failure, chronic lung disease, renal disease, diabetes mellitus with complications, diabetes without complications, 24-h vasopressor use, 24-h mechanical ventilation, mean hemoglobin, mean PH, and mean lactate levels. CI, confidence interval; HFNC, High-flow nasal cannula

The 28-day all-cause mortality rate among patients who received HFNC treatment was 18.63% (95 out of 510 patients), which was notably lower than the rate observed in the non-HFNC group, standing at 31.18% (159 out of 510 patients). Figure 2 presents the Kaplan–Meier curve illustrating the 28-day all-cause mortality based on HFNC utilization within the matched cohort. Both multivariate analysis (HR = 0.53; 95% CI = 0.41–0.69; *P* < 0.001) and univariate analysis (HR = 0.53; 95% CI = 0.41–0.69; *P* < 0.001) consistently indicated that the utilization of HFNC was significantly associated with a lower 28-day all-cause mortality rate.

### Subgroup analysis

**Fig. 3:**
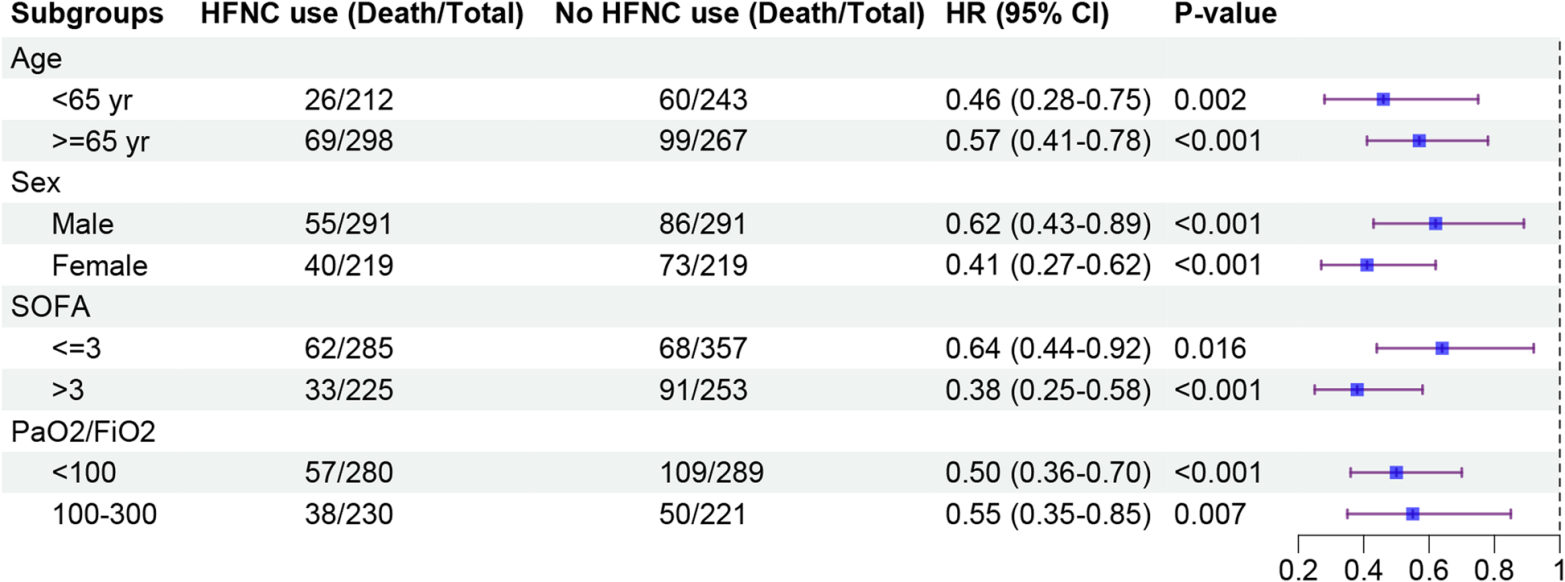
Analysis of subgroups for 28-day all-cause mortality in the matched cohort. The multivariable Cox proportional hazards model employed in this study incorporated adjustments for a range of factors. These factors included cerebrovascular disease, congestive heart failure, chronic lung disease, renal disease, diabetic complications, diabetes without complications, 24-h vasopressor use, 24-h mechanical ventilation, as well as mean hemoglobin, mean pH, and mean lactate levels. CI, confidence interval; HFNC, High-flow nasal cannula; HR, hazard ratio; SOFA, Sequential Organ Failure Assessment; PaO_2_/FiO_2_, arterial blood oxygenation index

The subgroup analysis within the matched cohort, as depicted in Fig. 3, explores the impact of HFNC use on the 28-day all-cause mortality rate across different patient characteristics. When stratifying by SOFA scores, patients with both lower (less than 4) and higher (4 or higher) severity showed significantly reduced mortality rates with HFNC use. Additionally, regardless of the baseline arterial oxygenation index (less than 100 or between 100 and 300), HFNC consistently improved patient survival. The upper limits of the 95% confidence intervals for all subgroups were below 1.00, highlighting a lower 28-day all-cause mortality rate associated with HFNC use across diverse patient profiles, reinforcing its potential benefit across varying levels of illness severity and oxygenation status.

### Secondary outcomes

In both univariate and multivariate analyses, the study investigated the impact of HFNC use on ICU mortality and in-hospital mortality rates. The ICU mortality rate in the HFNC group was notably lower at 14.90% (76 out of 510) compared to 25.29% (129 out of 510) in the group not using HFNC, as indicated by an OR of 0.52 (95% CI, 0.38 to 0.71; *P* < 0.001) in univariate analysis and an OR of 0.58 (95% CI, 0.41 to 0.82; *P* = 0.002) in multivariate analysis.

Similarly, the in-hospital mortality rate showed a significant difference between the HFNC and non-HFNC groups. The HFNC group exhibited a lower in-hospital mortality rate at 19.2% (97 out of 510) compared to 31.57% (161 out of 510) in the non-HFNC group. Univariate analysis yielded an OR of 0.51 (95% CI, 0.38 to 0.68; *P* < 0.001), and multivariate analysis confirmed this association with an OR of 0.54 (95% CI, 0.39 to 0.74; *P* < 0.001) (refer to Table 2).

**Table 2:** Association of HFNC use in the matched cohort with primary and secondary outcomes

Outcomes	Univariable analysis		Multivariable analysis	
	**HR/OR/MD (95% CI)**	***P*-value**	**HR/OR/MD (95% CI)**	***P*-value**
Primary outcome				
28-day all-cause mortality^a^	0.53 (0.41–0.69)	< 0.001	0.53 (0.41–0.69)	< 0.001
Secondary outcomes				
ICU mortality^b^	0.52 (0.38–0.71)	< 0.001	0.58 (0.41–0.82)	0.002
In-hospital mortality^b^	0.51 (0.38–0.68)	< 0.001	0.54 (0.39–0.74)	< 0.001
Length of ICU stay^c^	3.28 (2.61–4.02)	< 0.001	/	/
Length of hospital stay^c^	4.55 (3.32–5.80)	< 0.001	/	/

*CI* confidence interval, *HR* hazard ratio, *MD* median difference, *OR* odds ratio, In the multivariable Cox proportional hazards model, adjustments were made for cerebrovascular disease, congestive heart failure, chronic lung disease, renal disease, diabetes mellitus with complications, diabetes without complications, 24-h vasopressor use, 24-h mechanical ventilation, mean hemoglobin, mean PH, and mean lactate levels

^a^HR was calculated using Cox proportional hazards model

^b^OR was calculated using logistic regression model

^c^MD was calculated using HodgeseLehmann estimator

The analysis of the median length of stay in the ICU and overall hospitalization revealed significant differences associated with the use of HFNC. In the HFNC group, the median ICU length of stay was 9.47 days (IQR 5.665 to 15.395), compared to 5.55 days (IQR 2.855 to 10.655) in the non-HFNC group. For overall hospitalization, the median length of stay was 17.7944 days (IQR 11.026 to 26.6569) in the HFNC group and 11.70416667 days (IQR 7.09583 to 21.75312) in the non-HFNC group. Furthermore, the use of HFNC was found to be associated with a statistically significant increase in both ICU and overall hospitalization lengths. The MD in ICU stay was 3.28 days (95% CI, 2.61 to 4.02; *p* < 0.001), indicating a prolonged ICU stay in the HFNC group. Similarly, the MD for overall hospitalization was 4.547278 days (95% CI, 3.318823 to 5.803492; *p* < 0.001), suggesting an extended hospital stay associated with the use of HFNC.

## Discussion

The findings of the current study suggest a significant association between the use of HFNC in patients with sepsis and acute lung injury, and a notable reduction in the 28-day all-cause mortality rate. The consistency of this association across subgroup analyses enhances the reliability and generalizability of the study's conclusions. Moreover, the study reveals that HFNC usage is linked to decreased mortality rates not only within the ICU but also during in-hospital stays.

The association between HFNC therapy and mortality rates across various patient populations has been the focus of clinical investigations. Despite some conflicting findings, the overall trend suggests that HFNC may be clinically beneficial in specific contexts. As an example, a study covering 2,725 COVID-19 patients found that treatment with HFNC prior to endotracheal intubation may be associated with lower in-hospital mortality [21]. HFNC was also associated with a reduced risk of death in COVID-19 patients who were not mechanically ventilated within 6 h of admission, suggesting its effectiveness in the early stages of respiratory distress [22]. In addition, investigations targeting specific disease conditions have shown that HFNC treatment is also associated with reduced mortality in hypoxic respiratory failure due to idiopathic pulmonary fibrosis [23], further highlighting the applicability of HFNC in respiratory failure due to different etiologies. However, the results of studies addressing the impact of HFNC on mortality have been inconsistent. A meta-analysis of nine randomized controlled trials failed to reach a consistent conclusion on the effect of HFNC on mortality, despite its potential to reduce the need for tracheal intubation [7]. Another multicenter retrospective study found that HFNC had a lower mortality rate than other noninvasive ventilation modalities, but adjusted data showed that this difference was not significant [24]. The results of these studies may be affected by sample heterogeneity and potential confounders; therefore, more large-scale studies are needed to explore the impact of HFNC on mortality in different patient subgroups. Our study, which limited HFNC-treated patients and used propensity-matched scores to control for covariates and minimize confounding, showed that HFNC reduced the risk of death in patients with sepsis-related lung injury.

The HFNC treatment group exhibited a significant prolongation in both ICU and hospital length of stay as observed in the secondary outcomes. This finding is consistent with other studies. For instance, a study focusing on obese patients using HFNC indicated a significantly longer hospital and ICU stay in patients with a BMI ≥ 40 kg/m^2^ [25]. Similarly, a multicenter randomized controlled trial for chronic obstructive pulmonary disease demonstrated a significantly prolonged median hospital stay with HFNC compared to conventional oxygen therapy [26]. Additionally, Burnim's study also indicated that the use of HFNC in COVID-19 patients was associated with a longer length of hospital stay [22]. We believe that the HFNC group's prolonged length of stay may be due to a lower mortality rate, resulting in a longer survival time for these patients compared to the others.

In the management of patients with sepsis-associated lung injury, HFNC offers potential therapeutic benefits. Firstly, HFNC enhances oxygenation levels and reduces respiratory burden by delivering high-flow oxygen, thereby alleviating respiratory effort and fatigue. Secondly, HFNC provides a comfortable and well-tolerated alternative to traditional oxygen therapy, potentially enhancing patient compliance and treatment effectiveness. Additionally, HFNC therapy shows promise in preventing intubation and reducing complications associated with invasive ventilation methods, such as lowering the risk of ventilator-associated pneumonia and barotrauma. Lastly, by supporting early mobilization and rehabilitation efforts, HFNC may help expedite the recovery of critically ill patients and improve overall prognosis. These advantages highlight the significance of HFNC as a beneficial treatment choice for patients with lung injury.

HFNC enhances the physiological condition of patients with sepsis-related lung injury, leading to decreased mortality through various mechanisms. (1) Expiratory Positive Pressure Effect: The notable distinction between HFNC and traditional oxygen therapy lies in the delivery of a substantial 50–60 L/min airflow, accompanied by the warming and humidification of the gas [3, 27]. The airflow delivered by HFNC creates a positive pressure at the end of inhalation, helping to sustain alveolar recruitment, prevent alveolar collapse, and mitigate lung injury. (2) Reduced Work of Breathing: In a sepsis and HFNC study, alterations in respiratory pressure were gauged through esophageal pressure, revealing the efficacy of HFNC in diminishing respiratory drive among sepsis patients [28]. Additionally, the application of HFNC resulted in a noteworthy reduction in diaphragmatic electrical activity and the overall workload of breathing when compared to conventional oxygen therapy [29]. Relative to alternative non-invasive ventilation methods, HFNC exhibited superior comfort, fewer side effects, and a heightened level of patient cooperation [30]. (3) Alveolar Dilution and Cleansing Effect: The high-flow gas delivered by HFNC can dilute and cleanse secretions in the patient's airways, decreasing alveolar collapse and obstruction, enhancing ventilation and alveolar expansion [31].

Implementing HFNC therapy in patients with sepsis-induced acute lung injury requires consideration of various practical factors. HFNC therapy is suitable for patients with mild to moderate lung injury, but may not provide sufficient respiratory support for those with severe lung injury. We consider it more challenging to determine the cessation criteria for HFNC therapy compared to its initiation criteria. Besides utilizing conventional pulmonary function assessments, employing additional non-invasive tools such as ultrasound and electrical impedance tomography can assist in evaluating patients. Additionally, despite HFNC being a relatively safe treatment method, attention should be given to the prevention and management of complications such as nasal dryness, nosebleeds, and respiratory tract infections. High-quality specialized nursing can significantly reduce associated complications.

Our study has several limitations that warrant consideration. Firstly, the retrospective observational design introduces the possibility of residual confounders influencing clinical outcomes, despite our efforts in propensity score matching and conducting multivariate analysis. Due to the retrospective nature of the analysis, the present study lacks data on potential confounders such as the patients' previous lung function. Performing prospective randomized controlled trials or cohort studies can enhance the control of confounding variables and substantiate the observed benefits of HFNC therapy. Secondly, our investigation exclusively focused on the utilization of HFNC and did not delve into the analysis of alternative oxygen therapy options, such as conventional oxygen therapy, non-invasive positive pressure ventilation, and invasive mechanical ventilation. Thirdly, the criteria for determining when to discontinue HFNC remain unclear, with some studies suggesting that delayed tracheal intubation due to HFNC failure could potentially lead to increased mortality [32]. Finally, the absence of specific setting parameters for HFNC in the database limited our ability to perform subgroup analyses related to HFNC usage.

## Conclusions

In summary, our research broadens the scope of HFNC application, showcasing its efficacy in reducing 28-day all-cause mortality among critically ill patients with sepsis and acute lung injury. The incorporation of HFNC into clinical practice proves beneficial for disease management. We recommend early use of HFNC in patients with sepsis-associated acute lung injury. However, it is crucial to note that further validation through prospective studies is warranted to strengthen the reliability of this retrospective observation.

### Supplementary Information

**Additional file 1: Table S1.** Percentage of missing data. **Table S2.** Calculating the variance inflation factor for each variable in the entire cohort. **Table S3.** Calculating the variance inflation factor for each variable in the matched cohort. **Table S4.** Comparison of patient characteristics before and after propensity score matching. **Figure S1.** Equilibrium of distribution before and after propensity score matching.

**Additional file 2:** Specific code for calculating propensity score matching.

## Data Availability

All data from this study are publicly available in the MIMIC-IV database.

## Abbreviations

HFNC: High-flow nasal cannula
MIMIC-IV: Medical Information Mart in Intensive Care-IV
HR: Hazard ratio
CI: Confidence interval
OR: Odds ratio
ICU: Intensive Care Unit
CCU: Cardiovascular Care Unite
SOFA: Sequential Organ Failure Assessment
SQL: Structured query language
BMI: Body mass index
CRRT: Continuous Renal Replacement Therapy
VIF: Variance inflation factor
SD: Standard deviation
IQR: Interquartile range
MD: Median difference
SMD: Standard mean difference
COVID-19: Coronavirus Disease-2019
