# Supplementary material for: Association between high-flow nasal cannula use and mortality in patients with sepsis-induced acute lung injury: a retrospective propensity score-matched cohort study
Source: BMC Pulm Med. 2024 Apr 22;24:197. doi: 10.1186/s12890-024-03022-9 (PMC11036692; doi:10.1186/s12890-024-03022-9)

Table S1: Percentage of missing data

| **Variables** | **Missing count** | **Missing rate (%)** |
| --- | --- | --- |
| Lactate | 2411 | 23.1 |
| BMI | 2054 | 19.7 |
| Temperature | 759 | 7.3 |
| PH | 390 | 3.7 |
| WBC | 12 | 0.1 |
| Hemoglobin | 12 | 0.1 |
| Platelet | 12 | 0.1 |
| Respiratory rate | 7 | 0.1 |
| Heart rate | 3 | 0.0 |
| Mean arterial pressure | 3 | 0.0 |
| Age | 0 | 0.0 |
| Gender | 0 | 0.0 |
| Race | 0 | 0.0 |
| First care unit | 0 | 0.0 |
| SOFA | 0 | 0.0 |
| Cerebrovascular disease | 0 | 0.0 |
| Dementia | 0 | 0.0 |
| Rheumatic disease | 0 | 0.0 |
| Congestive heart failure | 0 | 0.0 |
| Chronic pulmonary disease | 0 | 0.0 |
| Diabetes with cc | 0 | 0.0 |
| Diabetes without cc | 0 | 0.0 |
| Renal disease | 0 | 0.0 |
| Mild liver disease | 0 | 0.0 |
| Severe liver disease | 0 | 0.0 |
| Malignant cancer | 0 | 0.0 |
| Antibiotic_6h | 0 | 0.0 |
| Antibiotic_24h | 0 | 0.0 |
| CRRT_24h | 0 | 0.0 |
| Vasoactive_24h | 0 | 0.0 |
| Ventilation_24h | 0 | 0.0 |
| PaO2/FiO2 | 0 | 0.0 |
| Charlson comorbidity index | 0 | 0.0 |
| The abbreviations are as same as Table 1 | |  |

Table S2: Calculating the variance inflation factor for each variable in the entire cohort

| **Variables** | **Variance inflation factor** |
| --- | --- |
| Charlson comorbidity index | 5.368233 |
| Age | 2.514527 |
| Severe liver disease | 1.795232 |
| Renal disease | 1.738508 |
| Mild liver disease | 1.684555 |
| Antibiotic_6h | 1.658878 |
| Antibiotic_24h | 1.63757 |
| Malignant cancer | 1.615743 |
| Lactate | 1.464058 |
| PH | 1.375844 |
| Congestive heart failure | 1.320266 |
| Heart rate | 1.307126 |
| Diabetes with cc | 1.305526 |
| Mean arterial pressure | 1.288436 |
| Respiratory rate | 1.28355 |
| SOFA | 1.280352 |
| Cerebrovascular disease | 1.279804 |
| Vasoactive_24h | 1.253315 |
| Platelet | 1.223084 |
| Chronic pulmonary disease | 1.218757 |
| Temperature | 1.21738 |
| CRRT_24h | 1.171685 |
| Hemoglobin | 1.15799 |
| Diabetes without cc | 1.14835 |
| PaO2/FiO2 | 1.137186 |
| First care unit | 1.116782 |
| BMI | 1.114884 |
| Ventilation_24h | 1.109628 |
| Gender | 1.086529 |
| WBC | 1.076514 |
| Dementia | 1.050779 |
| Rheumatic disease | 1.037588 |
| Race | 1.024081 |
| The abbreviations are as same as Table 1 | |

Table S3: Calculating the variance inflation factor for each variable in the matched cohort

| **Variables** | **Variance inflation factor** |
| --- | --- |
| Severe liver disease | 1.762547 |
| Mild liver disease | 1.648538 |
| Antibiotic_6h | 1.559614 |
| Antibiotic_24h | 1.552285 |
| Lactate | 1.527235 |
| Age | 1.460913 |
| PH | 1.44793 |
| SOFA | 1.33516 |
| Heart rate | 1.333406 |
| Vasoactive_24h | 1.330288 |
| Renal disease | 1.320577 |
| Respiratory rate | 1.266757 |
| Mean arterial pressure | 1.256928 |
| Platelet | 1.245146 |
| Temperature | 1.236412 |
| Diabetes with cc | 1.212205 |
| Hemoglobin | 1.210871 |
| Congestive heart failure | 1.205325 |
| CRRT_24h | 1.181403 |
| First care unit | 1.161314 |
| Cerebrovascular disease | 1.152871 |
| BMI | 1.143797 |
| Chronic pulmonary disease | 1.119512 |
| Gender | 1.118789 |
| Ventilation_24h | 1.11112 |
| Malignant cancer | 1.101892 |
| WBC | 1.086231 |
| Diabetes without cc | 1.072326 |
| Dementia | 1.072116 |
| PaO2/FiO2 | 1.060633 |
| Race | 1.059768 |
| Rheumatic disease | 1.04224 |
| The abbreviations are as same as Table 1 | |

|  |  |  |  |  |  |  |
| --- | --- | --- | --- | --- | --- | --- |

Table S4: Comparison of patient characteristics before and after propensity score matching

| **Variables** | | Before propensity score matching | | | | P-value | | After propensity score matching | | | | P-value | |
| --- | --- | --- | --- | --- | --- | --- | --- | --- | --- | --- | --- | --- | --- |
| Number of patients | | 9914 (No-HFNC) | | 510 (HFNC) | | | | 510 (No-HFNC) | | 510 (HFNC) | |  | |
| Age | | 65.71 (15.22) | | 65.97 (15.43) | | 0.703 | | 64.60 (16.59) | | 65.97 (15.43) | | 0.171 | |
| Gender | |  | |  | |  | |  | |  | |  | |
| Female | | 3773 (38.1) | | 219 (42.9) | | 0.03 | | 219 (42.9) | | 219 (42.9) | | 1 | |
| Male | | 6141 (61.9) | | 291 (57.1) | | | | 291 (57.1) | | 291 (57.1) | |  | |
| Race | |  | |  | |  | |  | |  | |  | |
| Black | | 616 ( 6.2) | | 18 ( 3.5) | | 0.034 | | 8 ( 1.6) | | 18 ( 3.5) | | 0.218 | |
| White | | 6619 (66.8) | | 344 (67.5) | | | | 343 (67.3) | | 344 (67.5) | |  | |
| Other | | 950 ( 9.6) | | 44 ( 8.6) | |  | | 51 (10.0) | | 44 ( 8.6) | |  | |
| Unknow | | 1729 (17.4) | | 104 (20.4) | | | | 108 (21.2) | | 104 (20.4) | |  | |
| First care unit | |  | |  | |  | |  | |  | |  | |
| Surgical ICU | | 1451 (14.6) | | 96 (18.8) | | <0.001 | | 85 (16.7) | | 96 (18.8) | | 0.604 | |
| Medical ICU | | 1808 (18.2) | | 102 (20.0) | | | | 116 (22.7) | | 102 (20.0) | |  | |
| CCU | | 4153 (41.9) | | 151 (29.6) | | | | 156 (30.6) | | 151 (29.6) | |  | |
| Other | | 2502 (25.2) | | 161 (31.6) | | | | 153 (30.0) | | 161 (31.6) | |  | |
| BMI | | 29.63 (7.74) | | 29.51 (8.63) | | 0.722 | | 29.77 (8.06) | | 29.51 (8.63) | | 0.615 | |
| SOFA | | 3.00 [2.00, 5.00] | | 3.00 [2.00, 5.00] | | 0.138 | | 3.00 [2.00, 5.00] | | 3.00 [2.00, 5.00] | | 0.21 | |
| Comorbidities | |  | |  | |  | |  | |  | |  | |
| Cerebrovascular disease | | 1496 (15.1) | | 58 (11.4) | | 0.025 | | 87 (17.1) | | 58 (11.4) | | 0.012 | |
| Dementia | | 233 ( 2.4) | | 16 ( 3.1) | | 0.324 | | 15 ( 2.9) | | 16 ( 3.1) | | 1 | |
| Rheumatic disease | | 332 ( 3.3) | | 13 ( 2.5) | | 0.391 | | 20 ( 3.9) | | 13 ( 2.5) | | 0.288 | |
| Congestive heart failure | | 2915 (29.4) | | 180 (35.3) | | 0.005 | | 142 (27.8) | | 180 (35.3) | | 0.013 | |
| Chronic pulmonary disease | | 2777 (28.0) | | 195 (38.2) | | <0.001 | | 147 (28.8) | | 195 (38.2) | | 0.002 | |
| Diabetes with cc | | 756 ( 7.6) | | 70 (13.7) | | <0.001 | | 29 ( 5.7) | | 70 (13.7) | | <0.001 | |
| Diabetes without cc | | 2452 (24.7) | | 103 (20.2) | | 0.023 | | 135 (26.5) | | 103 (20.2) | | 0.022 | |
| Renal disease | | 1835 (18.5) | | 102 (20.0) | | 0.432 | | 89 (17.5) | | 102 (20.0) | | 0.335 | |
| Mild liver disease | | 1469 (14.8) | | 76 (14.9) | | 1 | | 81 (15.9) | | 76 (14.9) | | 0.729 | |
| Severe liver disease | | 678 ( 6.8) | | 42 ( 8.2) | | 0.261 | | 39 ( 7.6) | | 42 ( 8.2) | | 0.817 | |
| Malignant cancer | | 1045 (10.5) | | 73 (14.3) | | 0.009 | | 56 (11.0) | | 73 (14.3) | | 0.132 | |
| Treatment | |  | |  | |  | |  | |  | |  | |
| Antibiotic_6h | | 6049 (61.0) | | 335 (65.7) | | 0.039 | | 315 (61.8) | | 335 (65.7) | | 0.216 | |
| Antibiotic_24h | | 7965 (80.3) | | 424 (83.1) | | 0.135 | | 435 (85.3) | | 424 (83.1) | | 0.39 | |
| CRRT_24h | | 347 ( 3.5) | | 22 ( 4.3) | | 0.397 | | 16 ( 3.1) | | 22 ( 4.3) | | 0.408 | |
| Vasoactive_24h | | 5954 (60.1) | | 247 (48.4) | | <0.001 | | 315 (61.8) | | 247 (48.4) | | <0.001 | |
| Ventilation_24h | | 4987 (50.3) | | 195 (38.2) | | <0.001 | | 227 (44.5) | | 195 (38.2) | | 0.049 | |
| Vital signs | |  | |  | |  | |  | |  | |  | |
| Heart rate (beats/min) | | 87.10 (15.53) | | 89.04 (16.56) | | 0.006 | | 89.25 (16.46) | | 89.04 (16.56) | | 0.834 | |
| Respiratory rate (bpm) | | 19.97 (4.18) | | 21.24 (4.17) | | <0.001 | | 21.09 (4.52) | | 21.24 (4.17) | | 0.594 | |
| Temperature (℃) | | 36.99 (0.68) | | 37.01 (0.54) | | 0.497 | | 36.98 (0.72) | | 37.01 (0.54) | | 0.477 | |
| Mean arterial pressure (mm Hg) | | 75.77 (9.29) | | 76.44 (9.59) | | 0.11 | | 75.62 (9.75) | | 76.44 (9.59) | | 0.173 | |
| Laboratory test | |  | |  | |  | |  | |  | |  | |
| WBC (10^9^/L) | | 12.95 (8.52) | | 13.76 (9.38) | | 0.038 | | 13.52 (10.45) | | 13.76 (9.38) | | 0.698 | |
| Hemoglobin (g/dl) | | 10.13 (1.60) | | 9.85 (1.89) | | <0.001 | | 10.21 (1.80) | | 9.85 (1.89) | | 0.002 | |
| Platelet (10^9^/L) | | 175.00 (96.53) | | 179.51 (98.52) | | 0.304 | | 178.31 (97.55) | | 179.51 (98.52) | | 0.846 | |
| PH | | 7.37 (0.07) | | 7.39 (0.07) | | <0.001 | | 7.36 (0.08) | | 7.39 (0.07) | | <0.001 | |
| Lactate (mmol/L) | | 2.17 (1.82) | | 1.97 (1.61) | | 0.013 | | 2.40 (2.18) | | 1.97 (1.61) | | <0.001 | |
| PaO_2_/FiO_2_ (mm Hg) | | 156.74 (68.11) | | 104.87 (49.37) | | <0.001 | | 103.65 (49.83) | | 104.87 (49.37) | | 0.693 | |

The abbreviations are as same as Table 1

Figure S1: Equilibrium of distribution before and after propensity score matching.

1. HFNC, before propensity score matching.
2. non-HFNC, before propensity score matching.
3. HFNC, after propensity score matching.
4. non-HFNC, after propensity score matching.


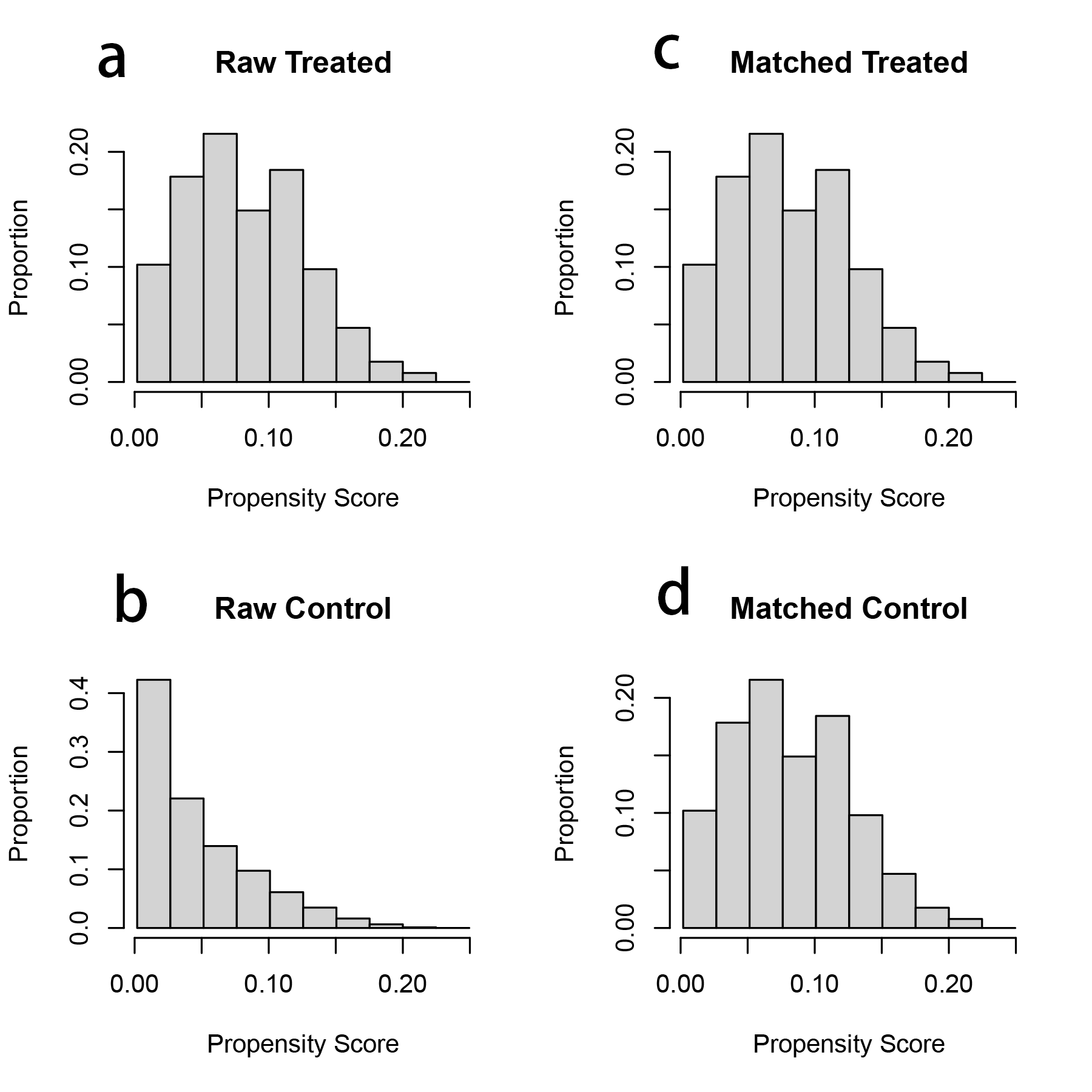

Supplement: Supplementary file 1 — Additional file 1: Table S1. Percentage of missing data. Table S2. Calculating the variance inflation factor for each variable in the entire cohort. Table S3. Calculating the variance inflation factor for each variable in the matched cohort. Table S4. Comparison of patient characteristics before and after propensity score matching. Figure S1. Equilibrium of distribution before and after propensity score matching. [file 12890_2024_3022_MOESM1_ESM.docx]
