# Supplementary material for: Association between high-flow nasal cannula use and mortality in patients with sepsis-induced acute lung injury: a retrospective propensity score-matched cohort study
Source: BMC Pulm Med. 2024 Apr 22;24:197. doi: 10.1186/s12890-024-03022-9 (PMC11036692; doi:10.1186/s12890-024-03022-9)
Supplement: Supplementary file 2 — Additional file 2: Specific code for calculating propensity score matching. [file 12890_2024_3022_MOESM2_ESM.docx]

Specific code for calculating propensity score matching

library(survival)

library(tableone)

library(MatchIt)

library(vroom)

setwd("C:/Users/songlijun/Desktop")

out<-vroom("C:/Users/songlijun/Desktop/shuju_all.csv",col_names = TRUE)

out$hfnc<-as.factor(out$hfnc)

out$gender<-as.factor(out$gender)

out$race<-as.factor(out$race)

out$first_careunit<-as.factor(out$first_careunit)

str(out)

vars<-c( "gender"," admission_age","race","first_careunit",

"sofa_score","BMI","pao2fio2ratio")

set.seed(1234)

psm<-matchit(hfnc ~ gender + admission_age + race+first_careunit+

sofa_score+BMI+pao2fio2ratio,

data = out,

method = "nearest",

ratio = 1,

replace=F,

distance = "logit",

caliper = 0.05 )

summary(psm)

matchdata<-match.data(psm)

View(matchdata)

plot(psm,type = "hist",interactive = FALSE)

print(matchdata,quote=T,noSpaces=T)

matchdata<-print(matchdata,

quote=F,

noSpaces=T,

printToggle=F)

write.csv(matchdata,file = "PSMnew.csv",row.names=F)
